# Antimicrobial and antioxidant potential of the silver nanoparticles synthesized using aqueous extracts of coconut meat (*Cocos nucifera L*)

**DOI:** 10.1038/s41598-023-43384-4

**Published:** 2023-09-27

**Authors:** Humaira Rizwana, Reem M. Aljowaie, Fatimah Al Otibi, Mona S. Alwahibi, Saleh Ali Alharbi, Saeed Ali Al asmari, Noura S. Aldosari, Horiah A. Aldehaish

**Affiliations:** 1 Department of Botany and Microbiology, College of Science, King Saud University, P.O. Box 22452, 11495 Riyadh, Saudi Arabia; 2 Department of Microbiology, Ministry of Health, Regional Laboratory, 14969 Riyadh, Saudi Arabia

**Keywords:** Biotechnology, Microbiology

## Abstract

Human pathogenic fungi and bacteria pose a huge threat to human life, accounting for high rates of mortality every year. Unfortunately, the past few years have seen an upsurge in multidrug resistance pathogens. Consequently, finding an effective alternative antimicrobial agent is of utmost importance. Hence, this study aimed to phytofabricate silver nanoparticles (AgNPs) using aqueous extracts of the solid endosperm of *Cocos nucifera* L, also known as coconut meat (Cm). Green synthesis is a facile, cost-effective and eco-friendly methods which has several benefits over other physical and chemical methods. The synthesized nanoparticles were characterized by UV–Vis spectroscopy, Fourier transform infrared spectroscopy (FTIR), X-ray diffraction analysis (XRD), field emission scanning electron microscopy (FE-SEM), transmission electron microscopy (TEM), and dynamic light scattering (DLS). The Cm-AgNPs showed a UV–Vis peak at 435 nm and were crystalline and quasi-spherical, with an average size of 15 nm. The FTIR spectrum displayed functional groups of phenols, alkaloids, sugars, amines, and carbonyl compounds, which are vital in the reduction and capping of NPs. The antibacterial and anticandidal efficacy of the Cm-AgNPs was assessed by the agar-well diffusion method and expressed as a zone of inhibition (ZOI). Amongst all the test isolates, *Staphylococcus epidermidis*, *Candida auris,* and methicillin-resistant *Staphylococcus epidermidis* were more susceptible to the NPs with a ZOI of 26.33 ± 0.57 mm, 19.33 ± 0.57 mm, and 18 ± 0.76 mm. The MIC and MFC values for Candida spp. were higher than the bacterial test isolates. Scanning electron microscopic studies of all the test isolates at their MIC concentrations showed drastically altered cell morphology, indicating that the NPs could successfully cross the cell barrier and damage the cell integrity, causing cell death. This study reports the efficacy of Cm-AgNPs against several Candida and bacterial strains, which had not been reported in earlier studies. Furthermore, the synthesized AgNPs exhibited significant antioxidant activity. Thus, the findings of this study strongly imply that the Cm-AgNPs can serve as promising candidates for therapeutic applications, especially against multidrug-resistant isolates of Candida and bacteria. However, further investigation is needed to understand the mode of action and biosafety.

## Introduction

Plant extracts and concoctions have played a vital role as phytomedicines and comprise secondary metabolites like alkaloids, terpenoids, and polyphenols^1,2^. Though crude extracts exhibit potent in vitro activity, the in vivo efficacy is reduced due to their solubility and reduced bioavailability, and this limitation can otherwise be enhanced through green synthesis^3^. Nanotechnology is a promising, efficient, and widely bourgeoning branch that has eradicated several limitations related to drug development, delivery, and bioavailability^4,5^. In fact, it has added several new innovative approaches and materials to the pharmaceutical, medical, and genetic fields, which has given a new face to medical and pharmaceutical scientific research^6–8^. Nanoparticles (NPs) are unique submicroscopic particles with at least one dimension less than 100 nm^9^, due to which their characteristics can be controlled^10^. Their exceptional properties like reactivity, strength, mobility, high surface-to-volume ratio, and solubility have enabled them to be the most appropriate material that can be incorporated in various applications in different sectors like agriculture, medicine, the environment, pharmaceutics, and engineering^11–13^. Besides, NPs possess astonishing interface effects that distinguish them from their corresponding bulk materials^14^.

Green synthesis of nanoparticles has occupied center stage in nanotechnology due to its eco-friendly, non-hazardous approach; besides, the materials used in green synthesis are easily available at a low cost^15,16^. Conversely, the synthesis of nanoparticles by physical and chemical methods has several disadvantages, such as the fact that it is difficult to control the size of the particles within the nanoscale limit, the high energy requirements, the high cost of fabrication, and most importantly, the release of toxic chemicals that have harmful effects on the environment^17,18^. Hence, the green synthesis method is preferred over the physical and chemical methods. The green synthesis method employs different microorganisms like algae^18^, fungi^19^, and bacteria cells^20^, or extracts from different parts of plants, including leaves^21,22^, shoots^23^, fruits, and fruit peels^24–26^. Green synthesis of nanoparticles using plants has several merits over other biological methods because it is a hassle-free, innocuous method that incorporates plant extracts and does not require sterile conditions or the arduous maintenance procedures that are required with other biogenic methods^27–29^. The aqueous plant extracts, with their vast variety of phytochemicals, function as stabilizers, capping agents, and reducers and can readily reduce metals to metal nanoparticles^29,30^. Metallic nanoparticles are unique due to their physiochemical and plasmonic properties^31^. Metals like zinc, gold, magnesium, iron, selenium, and silver synthesized using aqueous plant extracts have shown promising antimicrobial activity against pathogenic microorganisms^32^. However, silver is the most preferred metal due to its inherent antimicrobial and therapeutic properties. Green synthesized silver nanoparticles (AgNPs) have vast applications in food technology, pharmacology, microbiology, biotechnology, and medical fields^33^. The AgNPs synthesized using plant extract have fascinating physicochemical properties, are biocompatible and stable, and have proven to be efficacious against microorganisms^34^.

Human pathogenic fungi and bacteria pose a huge threat to human life, accounting for high rates of mortality every year. Another global concern is the upsurge in pathogenic microorganisms with antimicrobial multidrug resistance. The last two decades have witnessed a rapid rise in Candidiasis infections, especially in patients with chronic diseases, immunocompromised individuals, after organ transplants, and in nosocomial environments^35^. Although *Candida albicans* is most prevalent, non-albican Candida species (NAC) like *Candida auris*, *C. parapsilosis, C. famata, C. tropicalis, C. glabrata, and C. krusei* have been increasingly reported in health care personnel and immunocompromised individuals^36,37^. Candida spp. causes mucocutaneous and systemic infections, but the key challenge is the management of invasive candidemia (blood stream infection)^38^. Invasive candidemia is an emerging infection and accounts for more than 50,000 deaths every year worldwide^39^. Antifungal resistance and multidrug-resistant (MDR) Candida spp., further challenges and complicates the choice of antifungal therapy^38^. Presently, azoles, pyrimidine echinocandins, and polyene analogues are the major categories of antifungals used for treatment^40^. Nevertheless, the emergence of azole and echinocandin-resistant Candida species has raised concerns. Moreover, some oral antifungals cannot be administered for long durations as they are implicated with toxicities to vital organs, especially the liver^41,42^.

Similarly, bacterial pathogens with multidrug resistance are on the rise, and many have evolved to tolerate antibiotics^43^. Unfortunately, the critically important and high-priority bacteria with antibiotic resistance have increased in the past years and include *Staphylococcus aureus*, MRSA, *Escherichia coli*, *Klebsiella pneumoniae*, *Acinetobacter*, *Pseudomonas aeruginosa*, and *Enterobacter* spp**.**^44^. In 2019, antimicrobial resistance (AMR) was counted among the top ten global threats to human health by the World Health Organization^45^. Resistance to a single or multiple drugs by species of Candida and bacteria has further decreased the likelihood of effective treatment, raising concerns worldwide^35^. Therefore**,** finding safe and effective new treatments for resistant strains of Candida and bacteria is an important and pressing challenge that needs immediate attention. Herbal-based nanoformulations have garnered attention due to their enhanced bioavailability and solubility, sustained delivery, low toxicity, better stability, diminished side effects, limited accumulation in the organs, and improved therapeutic properties. Hence, we aimed to green synthesize AgNPs from the solid endosperm of coconut.

*Cocos nucifera* L is an economically important palm tree of the Arecaceae family. It is cultivated in tropical regions; Indonesia, India, Philippines, Thailand, and Sri Lanka are the major suppliers of coconut fruit to the world^46^. The fruit of this tree is a drupe and is commonly referred to as a coconut. It has an edible endosperm tissue, which consists of a solid white region called the coconut meat (Cm) and a liquid endosperm called the coconut water (Cw)^47^. The coconut meat is tender, and as it ripens, it gradually turns into a solid, hard endosperm. Coconut meat and water are rich sources of vitamins, phytochemicals, fatty acids (linoleic acid, lauric acid), sugars, amino acids (arginine, lysine), phytohormones, fiber, and minerals like manganese, potassium, and phosphorus^47–49^. Coconut water and coconut meat are used to treat oral mouth infections**,** mouth sores, and ulcers^49^. Earlier research has documented the antimicrobial, antioxidant, anti-inflammatory^49,50^, anti-viral^51^, anti-ulcerogenic^52^, and wound healing^53^ properties of coconut extracts. However, to the best of our knowledge, the bioactivity of AgNPs derived from Cm extract against species of Candida and bacteria has not been evaluated yet. Considering the meagre information available on green synthesized AgNPs of coconut meat and the potential benefits of several bioactive compounds present in Cm extract, this study aimed to synthesize AgNPs using fresh coconut meat, characterize them (UV–VIS, TEM, DLS, SEM, EDX, and FTIR), and evaluate their in vitro antifungal and antibacterial activities against some pathogenic species of Candida and bacteria. Besides, the antioxidant ability of Cm-AgNPs was also determined to evaluate their biomedical potential.

## Materials and methods

### Chemical compounds and culture media

Standard analytical-grade reagents and chemicals with 99% purity were used in the present study. Sabouraud dextrose agar (SDA), Sabouraud dextrose broth, distilled water, Nutrient agar (NA) agar/broth and ultra-pure water were procured from Sigma-Aldrich (Saint Louis, MO, USA). Silver nitrate (AgNO_3_) was purchased from Hi Media Labs (Mumbai, India). All the reagents and chemicals used in assays were procured from Sigma-Aldrich (Saint Louis, MO, USA).

### Preparation of aqueous coconut meat extract and synthesis of silver nanoparticles

Fresh coconut fruit (*Cocos nucifera*) was purchased from a local market in Riyadh. The plant material (coconut) used in the study was chosen keeping in mind the rules and regulations put forth by national and international guidelines and legislation and completely adhering to them. The fresh, soft white coconut meat (endosperm) was removed and roughly chopped.

The Cm pieces (20 g) were added to 200 mL of sterilized distilled water and heated at 60 °C for 20 min. The aqueous mixture of Cm was cooled and then filtered. The filtrate (Cm-extract) was centrifuged at 5000 rpm and was used for further work^15^. For the synthesis of silver nanoparticles, a reaction mixture comprising 45 ml of 1 mM aqueous silver nitrate solution and 5 ml (10 mg/ml) of milky white aqueous Cm extract was mixed. The reaction mixture was stirred continuously at 80 °C for 30 min, until the colour of the suspension changed. When the colour of the mixture changed to brown, it marked the formation of coconut meat silver nanoparticles (Cm-AgNPs). The reaction mixture was centrifuged for 30 min at 9,000 rpm and purified using distilled water. The process was repeated three times, and 140 mg of Cm-AgNPs were obtained. The synthesized Cm-AgNps were stored at 4 °C for further characterization and biological studies.

### Characterization of synthesized Cm-AgNPs

UV–vis spectroscopy validated the synthesis of Cm-AgNPs. The morphology and size were studied by TEM (TEM-JEOL JEM-Plus-1400, Tokyo, Japan). Around 200 NPs were counted. The average hydrodynamic size was determined by the Zeta sizer ultra-ZSU-3305 (Malvern Panalytical, Malvern, UK). The crystalline nature of Cm-AgNPs was validated by an X-ray diffractometer (Malvern, PANalytical-San Francisco, CA, USA). The X-ray tube and beam were operated at a voltage of 40 kV and 30 mA and scanned in the 2θ range. Fourier transform infrared spectroscopy was also conducted for both the extract and Cm-AgNPs to identify the functional groups. FTIR spectrometer (Thermo Scientific Model 6700, Waltham, Massachusetts, USA) and the KBr pellet method were used to obtain a spectrum, and the frequencies were collected at a resolution of 4 cm^−1^ in the range of 400–4000 cm^-1^. The EDS spectrum was captured at 30 kV on a field emission scanning electron microscope that was coupled with an energy dispersive X-ray detector (FESEM-EDAX-JSM-7610F-Japan). All the above-mentioned characterization techniques were carried out by following the instructions given by the manufacturer's.

### Pathogens

Both Candida spp. and bacterial strains were purchased from the American Type Culture Collection Centre (ATCC) and kindly provided by King Khalid University Hospital, Riyadh, Saudi Arabia. Several Candida strains were chosen for this study, which included *Candida parapsilosis* (ATCC-22019), *Candida krusei* (ATCC-6258), *Candida albicans* (ATCC-102310), *Candida glabrata* (ATCC-2950), and *Candida auris* (B21057582). The Candida strains were maintained on Sabouraud dextrose broth (SDB). The bacterial strains chosen were *Staphylococcus epidermidis* (ATCC 12228), Methicillin-resistant *Staphylococcus aureus*-MRSA (ATCC 43300), *Bacillus subtilis* (ATCC 6633), and *Staphylococcus aureus* (ATCC-25923). All the bacterial isolates were maintained on nutrient broth (NB).

### Anticandidal and antibacterial activity of Cm-AgNPs

The antimicrobial activity of Cm-AgNPs and aqueous Cm extract was assessed with the agar well diffusion method by CLSI (Clinical and Laboratory Standards Institute)^54^. Precisely, Candida spp. and bacterial test strains were cultured separately on SDA and NA for 24 h and incubated at 37 °C; a working suspension of test strains of Candida (2 × 10^4^ CFU/ml-colony forming unit) and bacteria (~ 1 × 10^7^ CFU/ml) that corresponded to a 0.5 Mac Farland was prepared. Briefly, 100 µL of the suspension was added separately to a new SDA/NA plate, and the suspension was evenly spread on the surface of the agar. After which, in each petri dish, four wells were punched, and each well was filled with either Cm-AgNPs (64 µg/ml), fluconazole (25 µg/mL), vancomycin (30 μg), 1 mM AgNO_3_, or an aqueous extract of coconut meat separately. The plates were incubated at 37 °C and observed after 24 h (bacteria) and 48 h (candida). The clear zone around each well was measured in mm. The assay was repeated and carried out in triplicate for each test isolate in a similar manner.

### Minimum inhibitory (MIC) and Minimum fungicidal/bactericidal (/MFCMBC) concentrations

The minimum inhibitory (MIC) concentrations for Candida and bacterial species were determined following the CLSI broth dilution method^55^. The assay was performed with two-fold serial dilutions of synthesized nanoparticles, fluconazole or vancomycin, separately, in the concentration range of 2–512 µg/mL with either Sabouraud dextrose broth for Candida spp. or Muller-Hinton broth for bacteria spp. Equal volumes of broth and suspension were added to the tubes. The broth contained 2 × 10^4^ CFU/mL of Candida spp. or 10^7^ CFU/mL of bacteria spp. Tubes were incubated for 24 h at 37 °C, and the minimum concentration that inhibited the visible growth of test isolates was regarded as the MIC. All the microbial strains were tested individually in a similar manner. About 50 µl of samples were drawn from tubes that exhibited no visible growth and were plated on either SDA/NA to ensure the observation was accurate. The negative control was spore suspension and SDA/NA broth without any addition (AgNPs), while the positive control was the tubes containing spore suspension, broth, and fluconazole/vancomycin. Minimum fungicidal concentration (MFC) and minimal bactericidal concentration (MBC) were determined by transferring 100 µL of broth from MIC tubes onto a respective sterile agar plate and incubating them as mentioned above. The concentration at which no growth was observed was assigned as MFC/MBC.

### Scanning electron microscopy (SEM)

A SEM study was done to identify the morphological effects induced by Cm-AgNPs on the test strains at their MIC concentrations. The selected suspensions at the MIC concentrations were centrifuged, and the cell sediment was taken for further processing. The samples were treated with glutaraldehyde (2.5%) and incubated overnight (4 °C). Further, after 24 h, the samples were washed three times with 0.1 M phosphate buffer solution (PBS-pH 7.4) for 20 min and subsequently treated (post-fixed) in 1% osmium tetroxide for an hour. Lastly, the samples were dehydrated with a series of ethanol solutions ranging from 60 to 100%. Post-dehydration, the samples were placed in a CPD (critical point dryer-liquid CO2) and coated with a very thin layer of gold. The samples were then examined, and microphotographs were obtained with a scanning electron microscope^30^.

### Phytochemical analysis of Cm-extract

To observe the common phytochemical constituents of Cm-aqueous extract, standard experimental procedures were conducted. The presence of the following phytochemicals was tested: saponins were detected by the foam test; alkaloids were analyzed by Dragendorff's reagents, Wagner's, and Mayer's reagents; tannins by Ferric Chloride´s test; flavonoids by the Shinoda alkaline reagent; and the Salkowski test was used to detect terpenoids^56–58^. The observations of these tests were indicated qualitatively as positive (+) or negative (−).

### Evaluation of Antioxidant Activity

The antioxidant property of Cm-AgNPs was tested with a DPPH-radical scavenging assay^15^. Varying concentrations in the range of 25, 50, 100, 150, 200, 250, and 300 μg/ml were prepared and mixed separately with 0.1 mM methanolic DPPH in order to obtain the final concentration. The mixture was agitated and incubated for 30 min (37 °C) in dark conditions. The absorbance was taken at 517 nm to monitor the decrease in DPPH concentrations. Ascorbic acid was used as a standard (control) and evaluated for its radical scavenging in a similar manner as aforementioned for AgNPs. A DPPH solution without any sample was used as a blank. The DPPH scavenging activity of Cm-AgNPs was calculated using ascorbic acid as the standard. The DPPH scavenging ability was calculated with the formula given below and expressed in %. Besides, the IC_50_ values of Cm-AgNPs and the ascorbic acid standard were calculated by implementing linear regression analysis, and a dose–response curve was plotted. The assay was conducted three times, and the values exhibited are an average of independent experiments run three times.

$${\text{DPPH scavenging assay }}\left( \% \right) = {\text{Cab }} - {\text{ TAb}}/{\text{CAb}} \times {1}00$$

where CAb is the absorbance of the control (without extract) and TAb is the absorbance of the test sample.

### 2,2′‐Azino‐bis(3‐ethylbenzothiazoline‐6‐sulphonic acid (ABTS) radical scavenging assay

The ABTS assay was conducted following the procedure cited earlier and with slight modifications^59,60^. In brief, the ABTS solution [7 mM] and potassium persulfate (2.45 mM) were combined together in equal proportions to prepare the free radical ABTS• + and incubated in dark conditions for 16 h. The resulting reaction mixture was diluted with ethanol to achieve 0.700 (± 0.03) at 734 nm. Further, this mixture was mixed with Cm-AgNPs at various concentrations (25, 50, 100, 150, 200, 250, and 300 μg/ml) and incubated for 6 min in the dark, and the absorbance was measured at 734 nm using ascorbic acid as a standard. The antioxidant activity was calculated following the standard formula aforementioned for the DPPH assay.

### Reducing power assay

The reducing power assay of Cm-AgNPs was determined by the previously reported protocol^61^. Briefly, 100 µl of various concentrations of Cm-AgNPs (25, 50, 100, 150, 200, 250, 300 μg/ml) were mixed with 400 µl of potassium ferricyanide (1% w/v) and 400 µl sodium phosphate buffer (0.2 mol/L-pH-6.6). The reaction mixture was incubated for 30 min at 50 °C, and subsequently, 400 µl of TCA (10% trichloroacetic acid) was added to the mixture. This mixture was then centrifuged (3000 rpm) for 10 min, and about 0.5 ml of supernatant was drawn from the mixture and mixed with 0.5 ml of deionized water and 0.1 ml of ferric chloride (0.1% w/v). The absorbance was taken at 700 nm. Both controls were tested in a similar manner as reported earlier.

### Hydroxyl radical (HO −) scavenging activity

The OH radical scavenging assay was performed by the method of Keshari et al.^61^ with slight modifications. About 0.075 ml of various concentrations of AgNPs were mixed with 0.45 ml (200 mM) of sodium phosphate buffer, 0.150 ml (10 mM) of FeSO4-EDTA, 0.15 ml H2O2 (10 mM), 0.15 ml (10 mM) of deoxyribose, and 0.525 ml of deionized water. The mixture was kept for 4 h in an incubator, and the reaction of the reaction mixture was ceased by adding 0.75 ml of 1% TBA and 0.75 ml of trichloroacetic acid (2.8%). The solution was then placed for 10 min in a boiling water bath. After cooling, the absorbance of the solution was recorded at 520 nm. Methanol served as a blank, while ascorbic acid was used as a standard. The percentage of hydroxyl radical scavenging activity was calculated using the aforementioned formula.

### Statistical analysis

All the values presented in table and figures in this study are experimental values from triplicates studies (SD). To evaluate the significant differences, Tukey’s HSD tests (p = 0.05) and analysis of variance (ANOVA) (p = 0.05) were conducted using Graph Pad Prism version 8.4.3.686 and OriginPro 2023.

## Results

### Green synthesis and characterization of Cm-Ag-NPs

**Figure 1:**
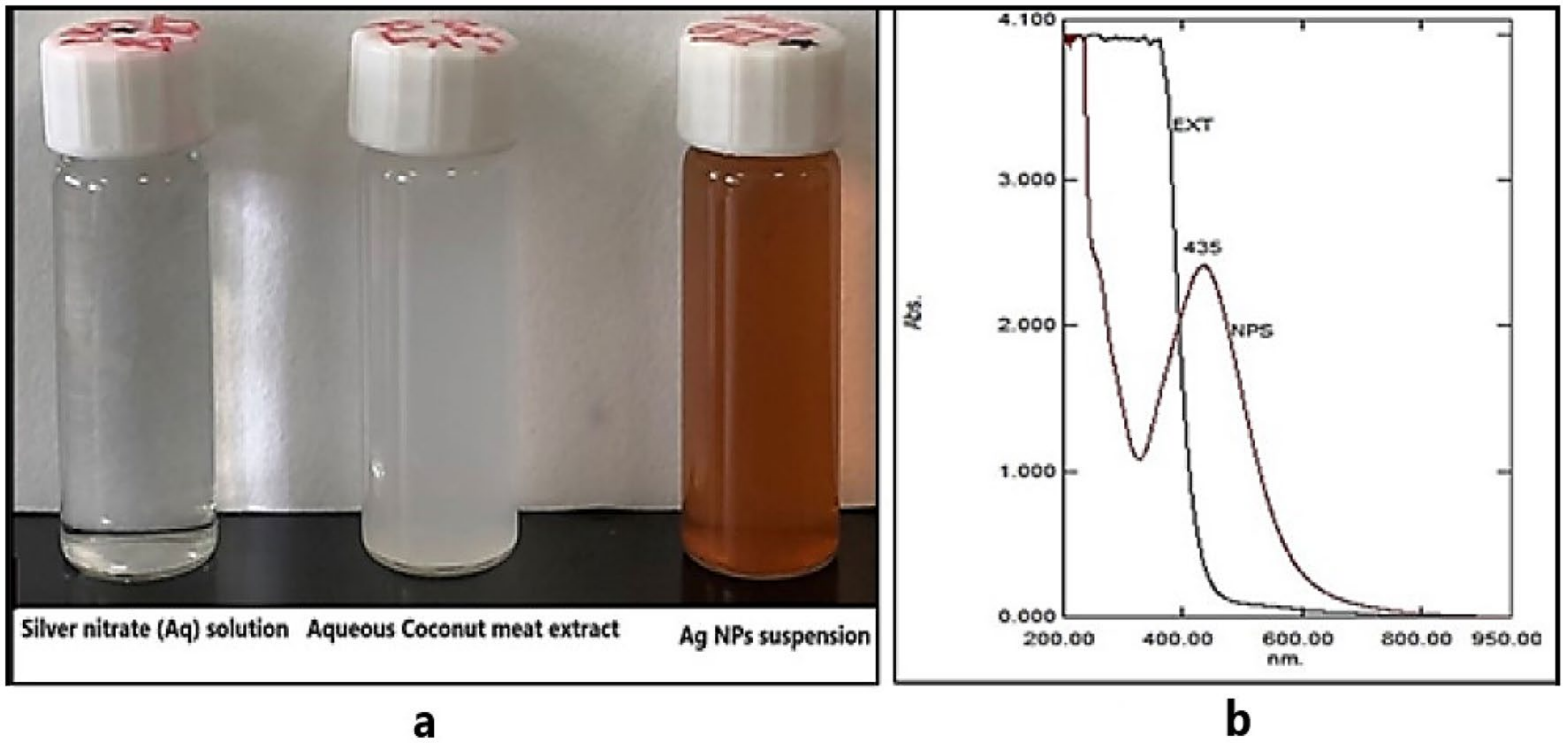
(**a**) Synthesis of silver nanoparticles from aqueous Coconut meat extract (**b**) UV–Vis spectrum of Cm-AgNPs at 435 nm.

**Figure 2:**
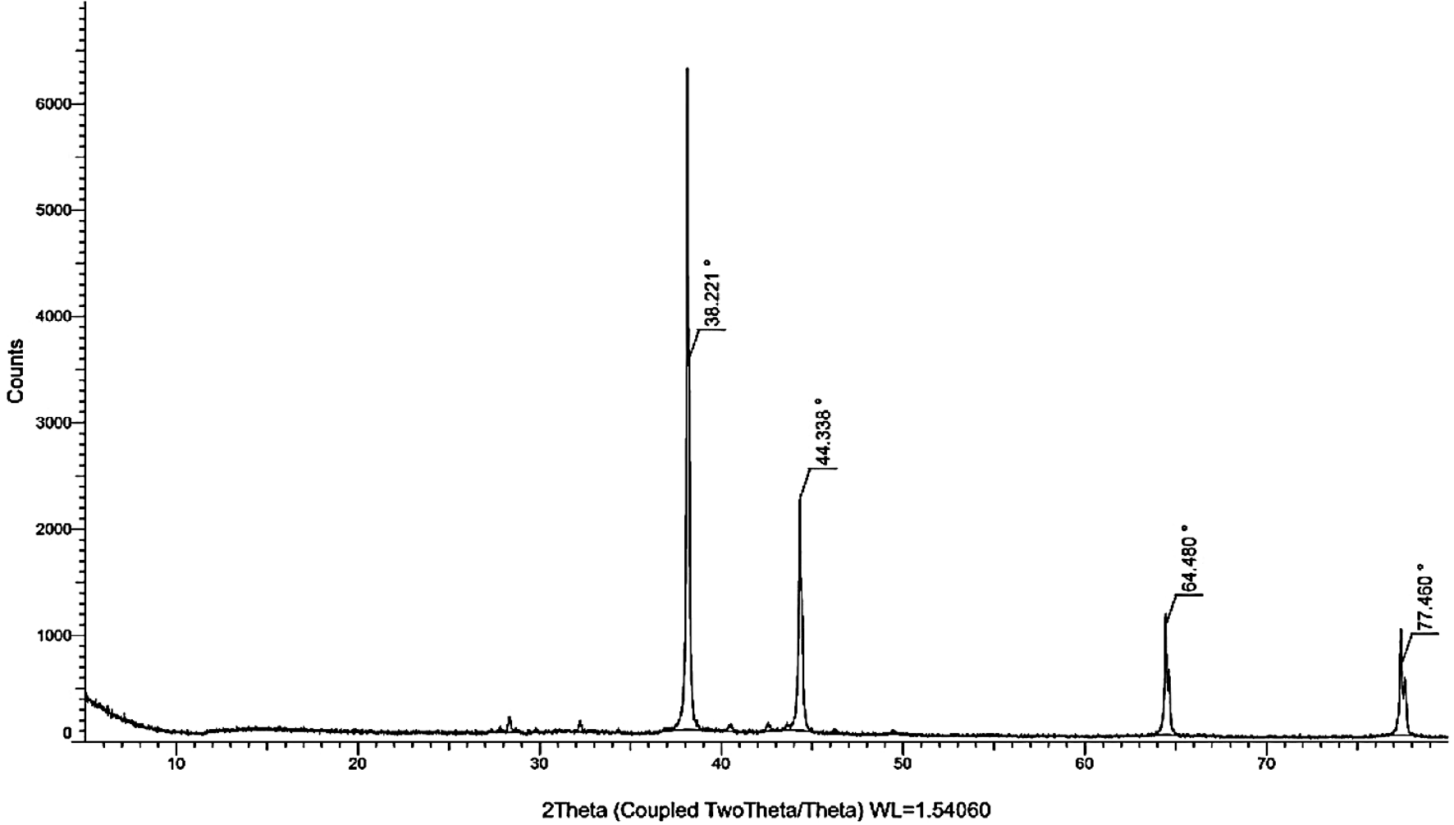
XRD pattern of Cm-AgNPs.

**Figure 3:**
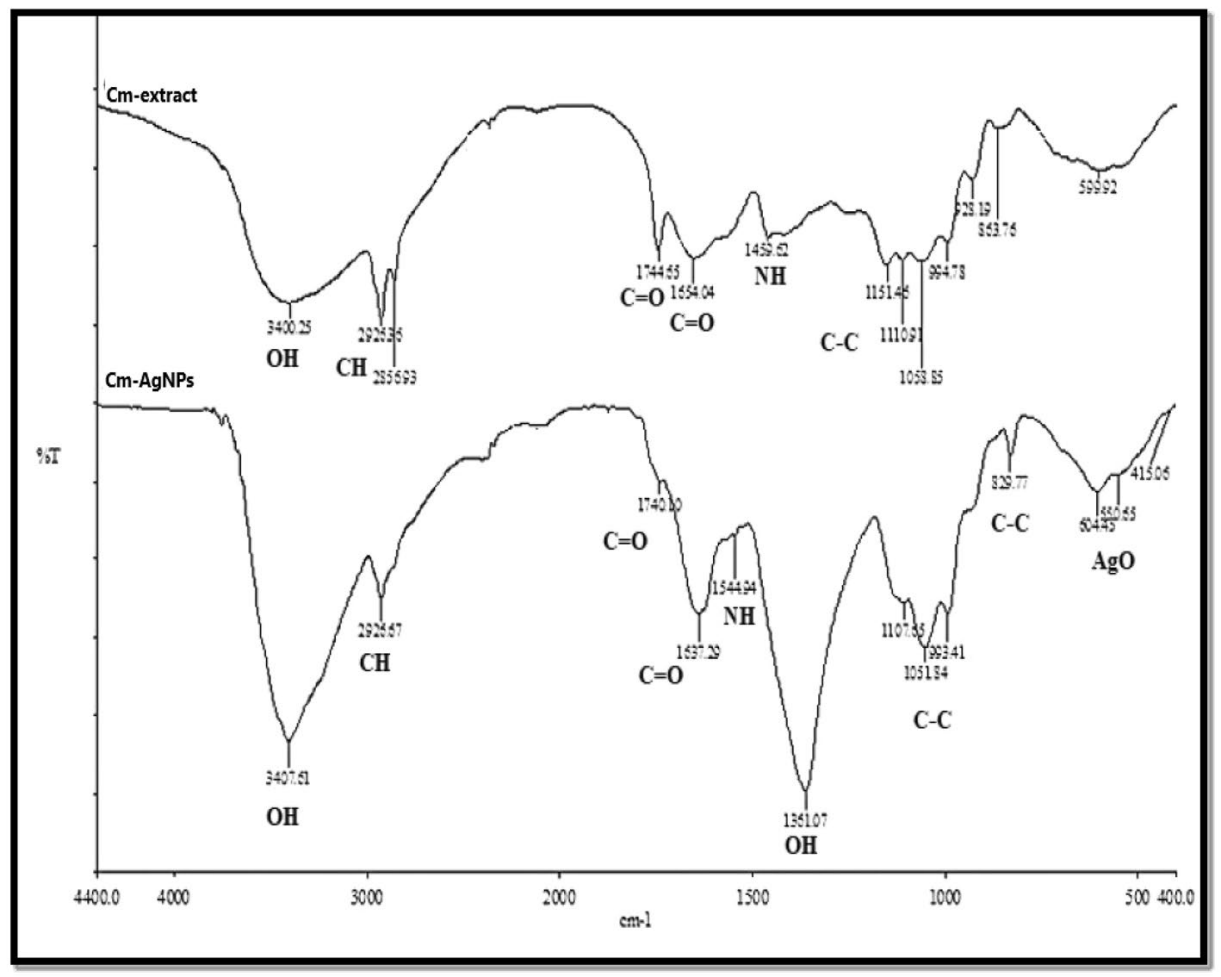
Peaks related to functional groups of various phytochemicals present in aqueous extracts of Cm and Cm-AgNPs are depicted in FT-IR spectrum.

The as-obtained Cm aqueous extract was milky white. The addition of Cm extract to a 1 mM AgNO3 solution showed an apparent transformation in colour from milky white to brown, validating the formation of the desired Ag-NPs (Fig. 1a). The UV–Vis spectroscopy of the Ag-NPs manifested a broad surface plasmon resonance peak at 435 nm on the spectrum, further corroborating the successful synthesis of Cm-derived Ag-NPs (Fig. 1b). The XRD pattern of Cm-AgNPs is shown in Fig. 2. The diffractogram pattern demonstrates peaks (2θ) at positions 38.14°, 44.32°, 64.46°, and 77.41°, which is a reflection of the planes at 111, 200, 220, and 311 corresponding to Miller’s indices. Additionally, the XRD patterns did not show any impurities. The FTIR spectra of Cm extract and Cm-Ag-NPs were captured in the wavelength range of 400–4000 cm^−1^ and are presented in Fig. 3 and Table 1. All the significant functional groups, including carbonyl, hydroxyl, and amines, are quite evident in the spectra of both the extract and Cm-AgNPs. The spectrum of Cm-extract showed prominent peaks at 3400 cm^-1^, 2926 cm^-1^,1654 cm^-1^ 1744 cm^-1^, and 1459 cm^-1^ which are relatable to O–H, C–H, and C=O, C=C, and NH stretching and bending vibrations (Table 1). The presence of strong bands at ∼1654 cm^−1^ and ∼1459 cm^−1^ on the spectra of the extract is a clear indication of the presence of amide (I, II) proteins. The spectrum of fabricated Cm-AgNPs was quite identical to the spectrum of Cm-extract (Fig. 3-Cm-AgNPs, Table 1), except that some peaks had shifted to lower frequencies (1740 cm^−1^, 1637 cm^−1^, 1107 cm^−1^ and 1051 cm^−1^). Besides, new peaks at 1544 cm^-1^,1361 cm-^1^ , 604 cm^−1^ and 550 cm^−1^ were also evident on the spectrum.

**Table 1:** Functional groups present in the Cm extract and Cm-AgNPs.

FTIR peaks(cm^−1^) Cm-extract Cm-AgNPs		Functional groups assigned
3400	3407	Symmetric and asymmetric OH-stretching vibrations of hydrogen bonded hydroxyl groups^16,19^
2926	2856	Asymmetric and symmetric vibrations of CH, –CH2 and NH^15,29^
1744	1740	C=O stretching vibrations of carbonyl^7,15^
1654	1637	Stretching vibrations of the amide bonds(I, II) of C=O (24,6)
1459	1544	NH bending vibrations of amides^60,69^
1110 1058	1107 1051	C–O and C–O–C stretching vibrations of primary and secondary alcohols^31,71^

**Figure 4:**
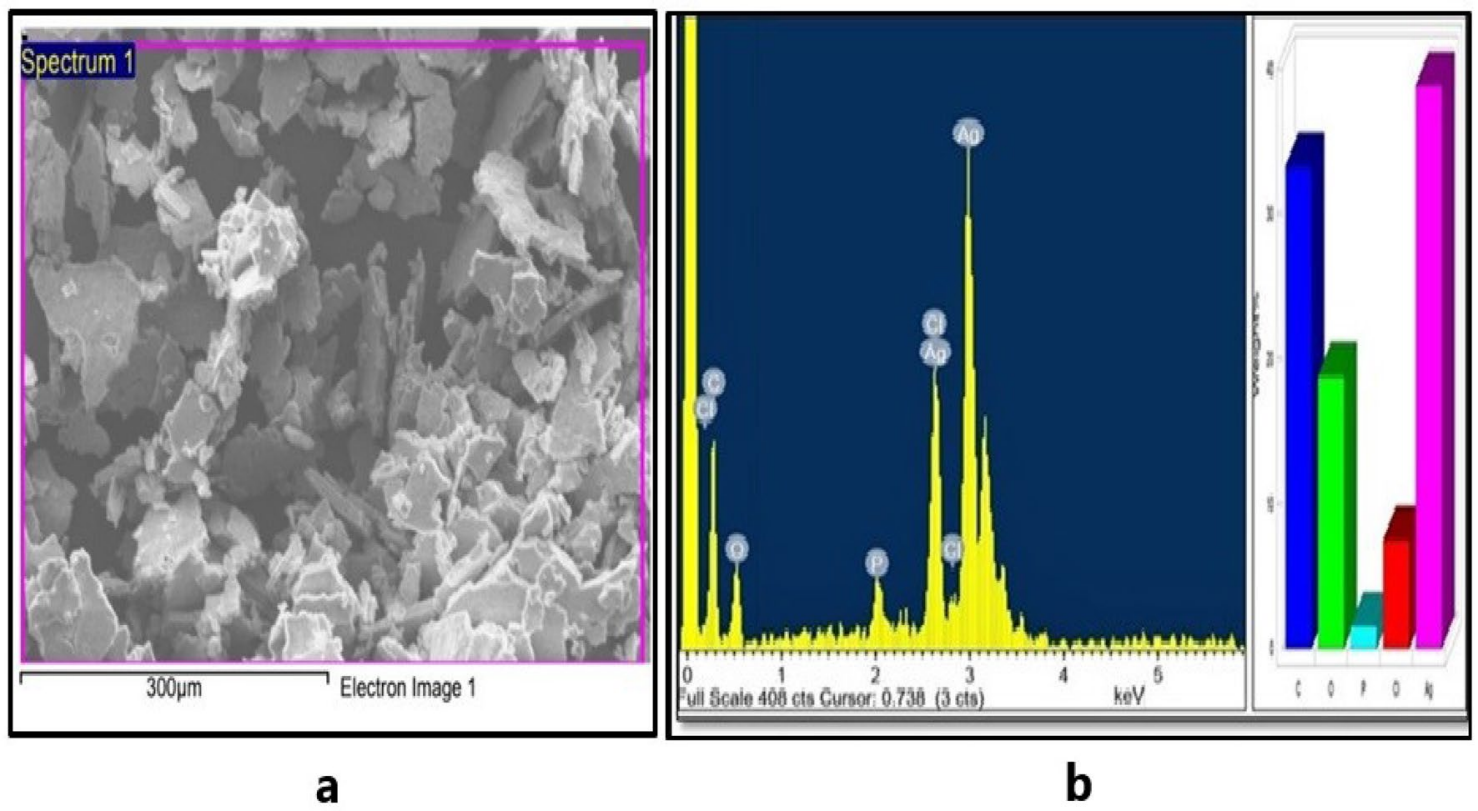
(**a**) FESEM microphotograph and (**b**) EDS spectrum of elemental analysis of Cm-AgNPs prepared with aqueous coconut meat extract.

**Figure 5:**
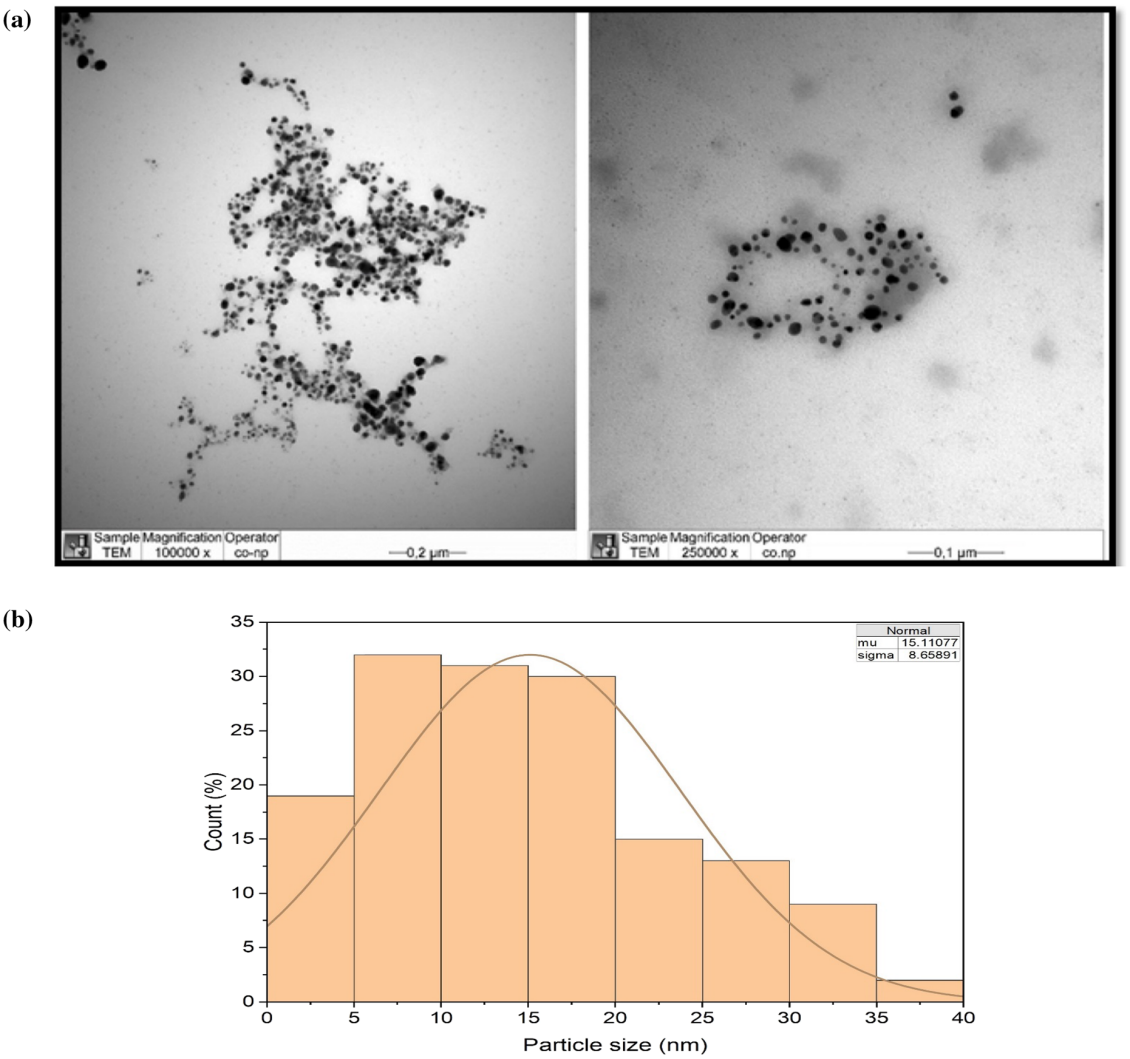
(**a**) TEM microphotographs showing morphology of Cm-AgNPs in two different magnifications. (**b**) Histogram of the size distribution (Diameter) of Cm-AgNPs based on TEM images.

**Figure 6:**
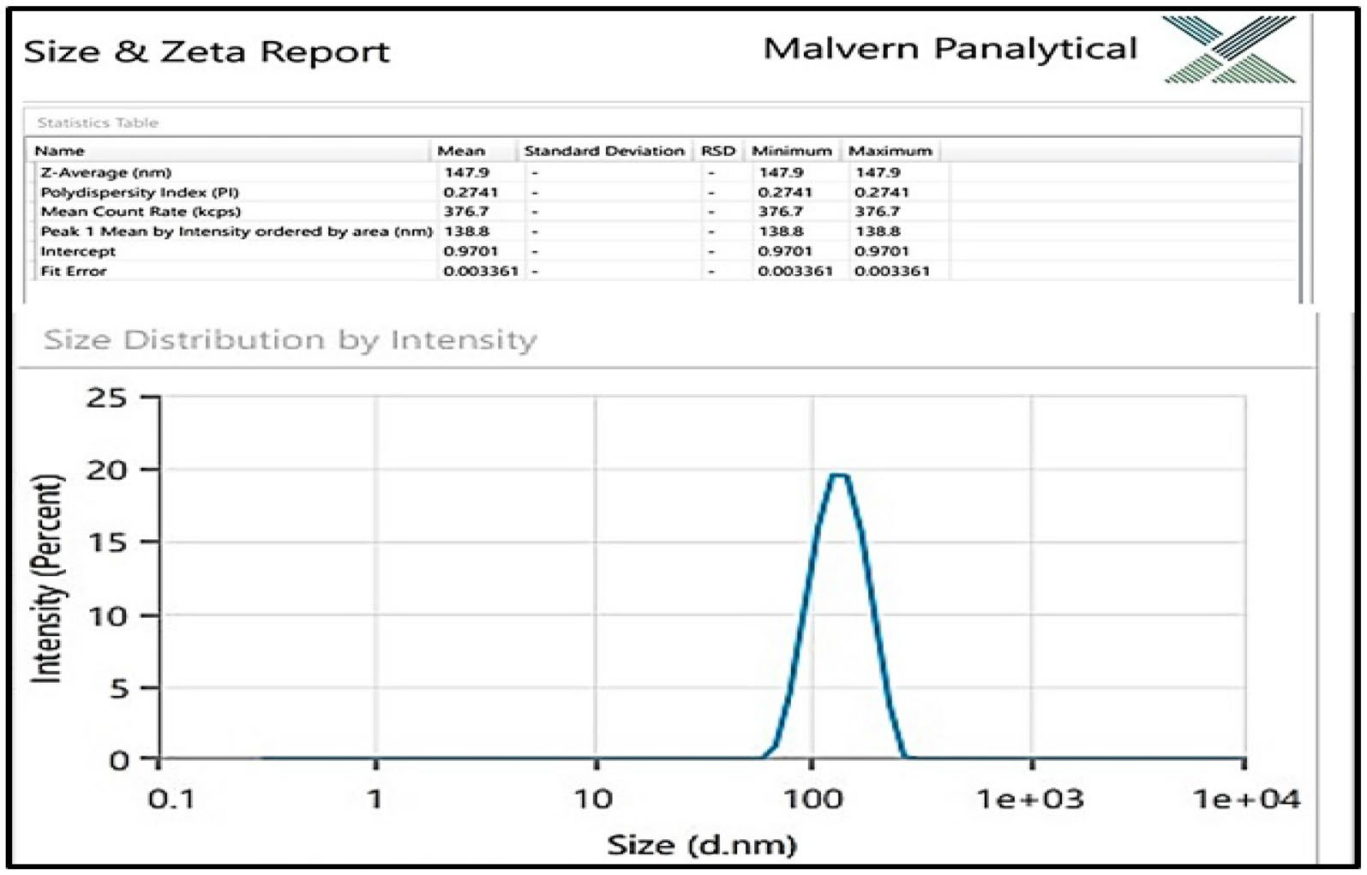
Dynamic light scattering image depicting (Z average) hydrodynamic size distribution (intensity weighted) of Cm-AgNPS.

The FESEM-EDS spectrum in Fig. 4a,b depicts the scanning electron microphotograph of Cm-AgNPs and the elemental composition of the Cm-AgNPs. The dry sample of NPs displayed a distinctive peak related to the silver at 3 keV and 2.6 keV**,** indicating the presence of elemental silver and confirming the synthesis of Cm-AgNPs. Silver was the major element (40%), while the other signals arose from the typical absorption of carbon (33%), chlorine (7.9%), oxygen (17%), and phosphorus (3.3%). Transmission electron microphotographs are presented in Fig. 5a. The images clearly show dense, relatively spherical, well-dispersed nanoparticles. The size of NPs varied from 2.01 to 38.76 nm, and the average size was 15.1 nm (Fig. 5b). The DLS hydrodynamic size of the nanoparticles was 147 nm with a PDI (polydiversity index) of 0.27 (Fig. 6).

### Anticandidal and antibacterial activity of Cm-AgNPs

**Figure 7:**
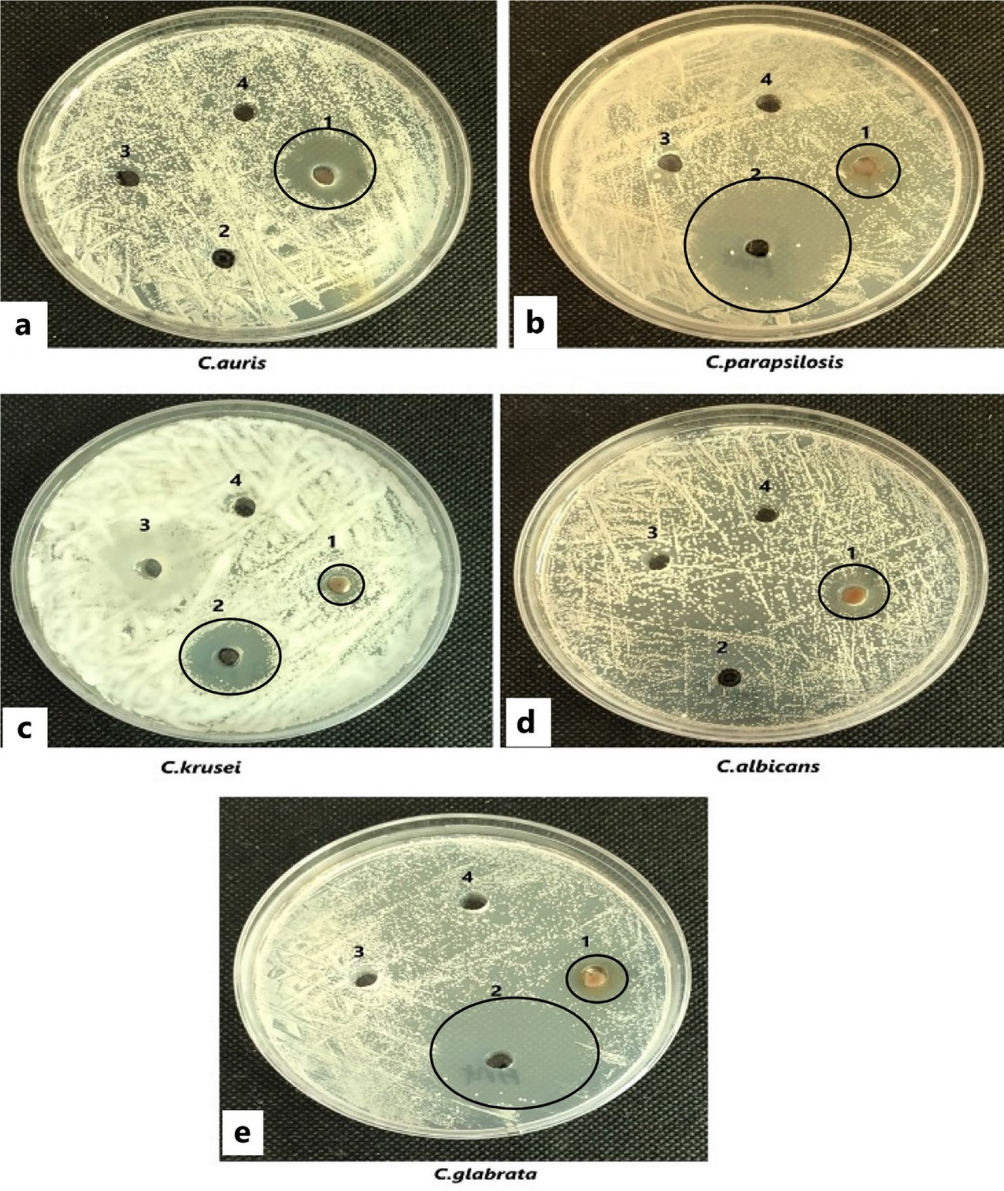
Anticandidal activity of silver nanoparticles synthesized from aqueous extracts of Coconut meat determined by well diffusion method against (**a**) *Candida auris* (B21057582), (**b**) *Candida parapsilosis* (ATCC-22019), (**c**) *Candida krusei* (ATCC-6258), (**d**) *Candida albicans* (ATCC-102310), (**e**) *Candida glabrata* (ATCC-2950). The numbers are designated as follows (1) Cm-AgNPs (2) Flucanazole (3) Cm-aq extract (4) aqueous AgNO_3_ solution.

**Figure 8:**
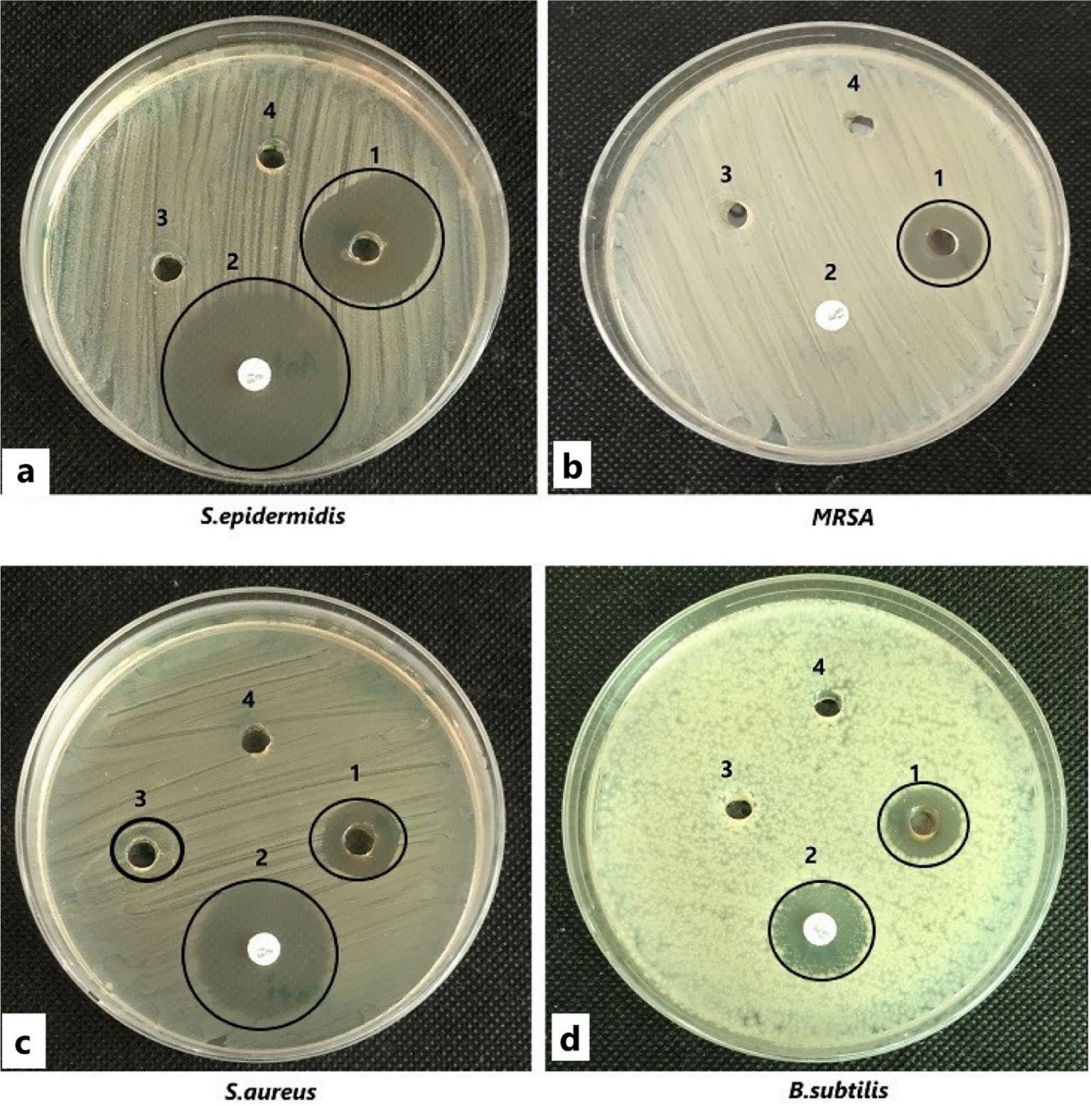
Antibacterial activity of silver nanoparticles synthesized from aqueous extracts of Coconut meat determined by well diffusion method against (**a**) *Staphylococcus epidermidis* (ATCC 12228), (**b**) MRSA-Methicillin-resistant *Staphylococcus aureus* (ATCC 43300), (**c**) *Staphylococcus aureus* (ATCC-25923) (**d**) *Bacillus subtilis* (ATCC 6633). The numbers are designated as follows (1) Cm-AgNPs (2) Vancomycin (30 µg) (3) Cm-aq extract (4) aqueous AgNO_3_ solution.

The in vitro anticandidal and antibacterial activity of the Cm-Ag-NPs against five species of Candida and four species of gram-positive bacteria was assessed by the agar-well diffusion method, and the inhibitory concentration was evaluated by the two-fold microdilution method. The growth inhibition was expressed as the clear zone of inhibition (ZOI-mm) around each well. The Cm-Ag-NPs inhibited all the Candida species used in this study (Fig. 7, Table 2). However, maximum inhibition was demonstrated by *C. auris* (19.33 mm), and the least inhibited with the smallest ZOI was *C. krusei* (10 mm). Fluconazole served as a positive control. Aqueous extract and silver nitrate did not inhibit any of the tested isolates; hence, they are not shown in Table 2. The findings of antibacterial activity show the robust growth inhibitory activity of Cm-AgNPs against all the tested bacterial isolates, as the ZOIs were larger than Candida spp. (Figs. 8 and Table 3). The largest ZOI was shown by *S. epidermidis* (26 mm), followed by MRSA (18 mm). These findings clearly indicate the strong susceptibility of bacterial isolates to the synthesized NPs. The positive control (Vancomycin 30 μg) showed strong inhibition against all the bacterial isolates except MRSA (Table 3). The MIC and MFC values of Cm-AgNPs against Candida spp. ranged between 16 and 256 µg/ml and 64–512 µg/ml (Table 4), while the MIC and MBC values for bacterial isolates were 4–16 µg/ml and 8–64 µg/ml, respectively (Table 5).

**Table 2:** Anticandidal activity of Cm-Ag-NPs as determined by well diffusion method. The values shown in the table are means of three replicates (± SD). Analysis of variance (ANOVA) and Tukey’s HSD were run to determine significant difference in means (p ≤ 0.05). NI-not inhibited.

Treatment	Zone of inhibition (mm)				
	*Candida auris*	*Candida parapsilosis*	*Candida krusei*	*Candida albicans*	*Candida glabrata*
AgNPs	19.33 ± 0.57	11.83 ± 0.28	10.00 ± 1.00	12.83 ± 0.57	11.00 ± 0.5
Flucanozole	NI	38.33 ± 1.00	19.33 ± 0.57	NI	39.66 ± 0.00

**Table 3:** Antibacterial activity of Cm-AgNPs as determined by well diffusion method.

Treatment	Zone of inhibition (mm)			
	*S. epidermidis*	*MRSA*	*S. aureus*	*B. subtilis*
Cm-AgNPs	26.33 ± 0.57	18.16 ± 0.76	16.33 ± 0.57	15.50 ± 0.5
Cm-aq extract	NI	NI	9.33 ± 0.76	NI
Vancomycin 30 µg	33.33 ± 0.76	NI	29.00 ± 1.00	18.50 ± 0.86

The values shown in the table are means of three replicates (± SD). Analysis of variance (ANOVA) and Tukey’s HSD were run to determine significant difference in means (p ≤ 0.05). NI-not inhibited.

**Table 4:** Minimum inhibitory and minimum fungicidal concentration of Cm-AgNPs on fungal test strains.

Concentration (µg/ml)				
Fungal strains	Cm-Ag-NPs		Flucanozole	
	MIC	MFC	MIC	MFC
*Candida auris*	16	64	32	64
*Candida parapsilosis*	128	128	2	4
*Candida krusei*	128	512	32	64
*Candida albicans*	64	64	4	8
*Candida glabrata*	128	256	4	8

**Table 5:** Minimum inhibitory and minimum bactericidal concentration of Cm-AgNPs on bacterial test strains.

Concentration (µg/ml)				
Bacterial strains	Cm-Ag-NPs		Vancomycin	
	MIC	MFC	MIC	MFC
*S. epidermidis*	4	8	2	4
MRSA	8	32	32	64
*S. aureus*	16	32	2	4
*B. subtilis*	16	64	4	8

### Scanning electron microscopy (SEM)

**Figure 9:**
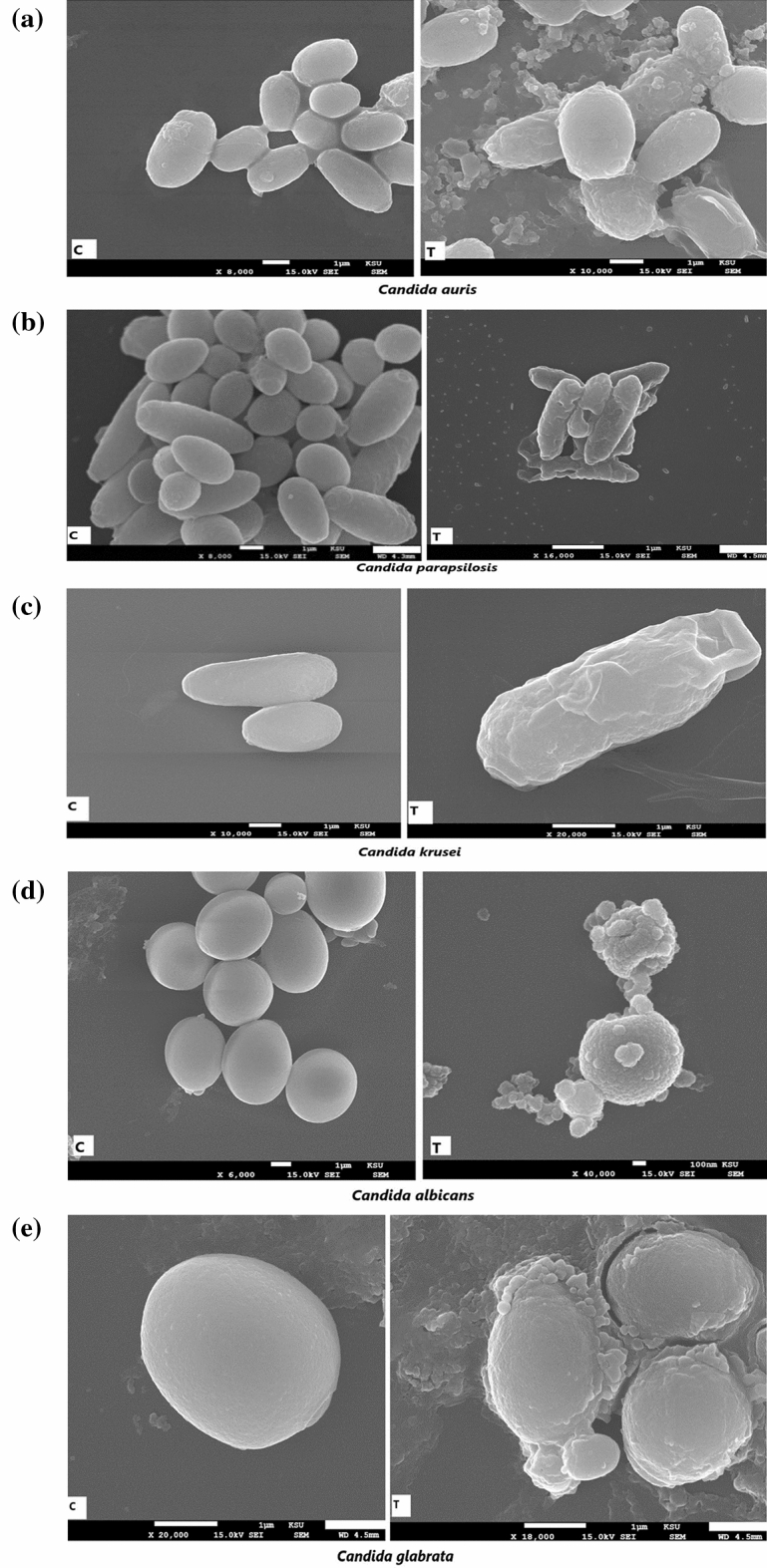
Scanning electron microphotographs of (**a**) *Candida auris*, (b) *C.parapsilosis*, (**c**) *C.krusei*, (**d**) *C. albicans,* and (**e**) *C. glabrata* at their respective MIC concentrations show altered morphology with corrugated cell surfaces and cell leakage in comparison to untreated cells which exhibit well-defined, regular and smooth cell margins. C-control-untreated, T-treated with Cm-AgNPs.

**Figure 10:**
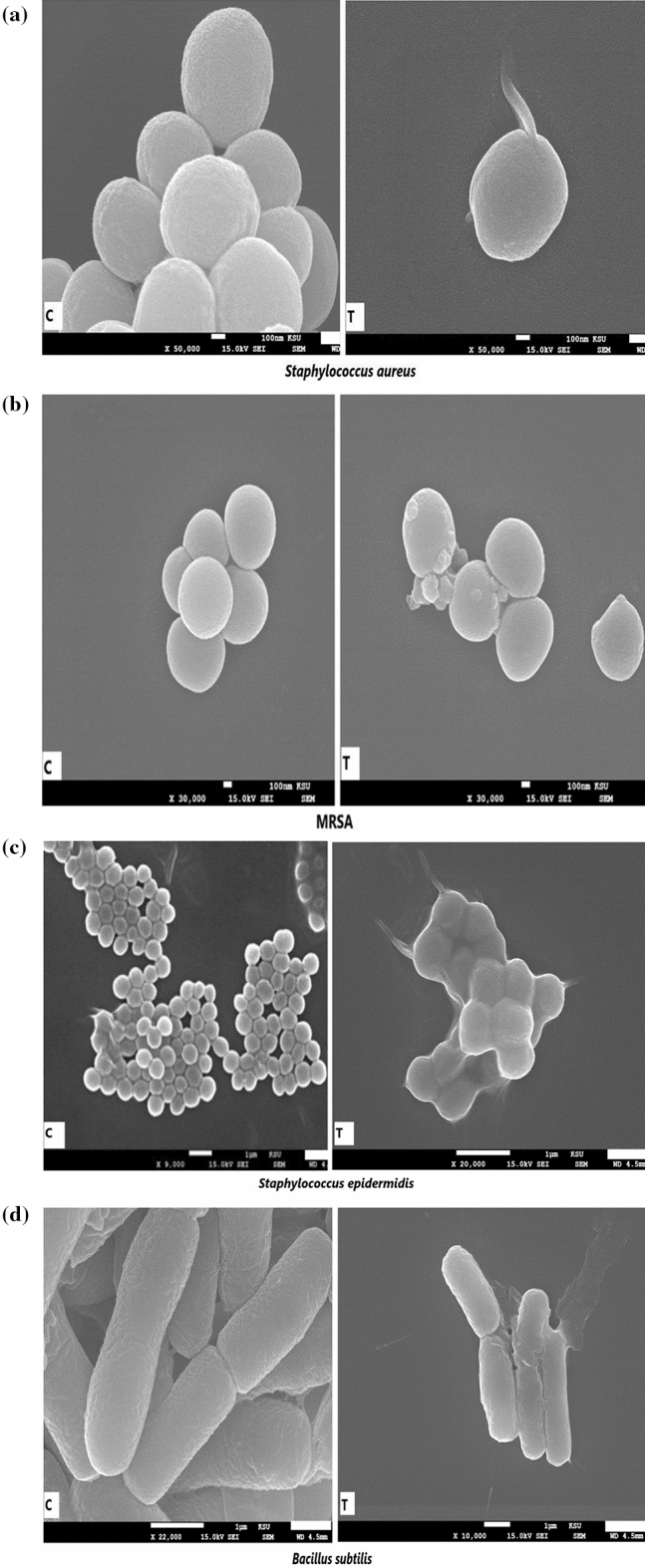
Scanning electron microphotographs of (**a**) *Staphylococcus aureus*, (**b**) *MRSA-Methicillin-resistant Staphylococcus aureus*, (**c**) *Staphylococcus epidermidis,* and (**d**) *Bacillus subtilis* show severely damaged and deformed cells (MIC concentrations) in comparison to the control (not treated). C-control-untreated, T-treated with Cm-AgNPs.

The morphological alterations induced by Cm-AgNPs on all the test isolates (Candida and bacteria) used in this study were scanned with SEM and captured on microphotographs (Figs. 9, 10). The untreated samples (controls) of both bacteria and Candida were also scanned for comparison with the treated sample. The samples at their MIC concentrations were used for SEM studies. The untreated samples of all bacterial and Candida species are presented as "C" in all the figures, while the treated samples are T." The control (untreated) samples of all test strains displayed regular cell margins, a smooth surface, and intact cell structure. Whereas the samples treated with Cm-AgNPs showed deformed cells with totally altered cell morphology (Fig. 9, 10a–e). Figure 9a portrays mutilated cells of *C. auris* surrounded by NPs. Figure 9b reveals the severely damaged *C. parapsilosis* cells with hollow regions and cavities, indicating cell death. The C. *krusei* cell shown in the figure was totally distorted and had lost its original shape and cell morphology Fig. 9c. *C. albicans* cells were surrounded by NPs; deep disc-like depressions and cell leakage are quite notable in the microphotograph Fig. 9d. *C. glabrata* cells were damaged and misshapen Fig. 9e. In the same manner, the microphotographs of bacterial cells, including *S. aureus,* MRSA, *S. epidermidis, and B. subtilis,* treated with NPs illustrate discernible morphological alterations, including uneven cell surfaces, holes, and cell leakage (Figs. 10a–d). In general, the cells treated with AgNPs had a corrugated cell surface with nanoparticles either adhering to or surrounding the treated cells.

### Phytochemicals

The phytochemicals present in the aqueous extract of coconut meat were analyzed following the standard tests. Alkaloids, saponins, tannins, and terpenoids were among the phytochemicals found in the extract of Cm (Table 6).

**Table 6:** Phytochemical analysis of Cm-extract.

Phytochemicals	Cm-aqueous extract
Alkaloids	+
Tannins	+
Saponins	+
Flavonoids	−
Terpenoids	+

### Antioxidant activity

**Figure 11:**
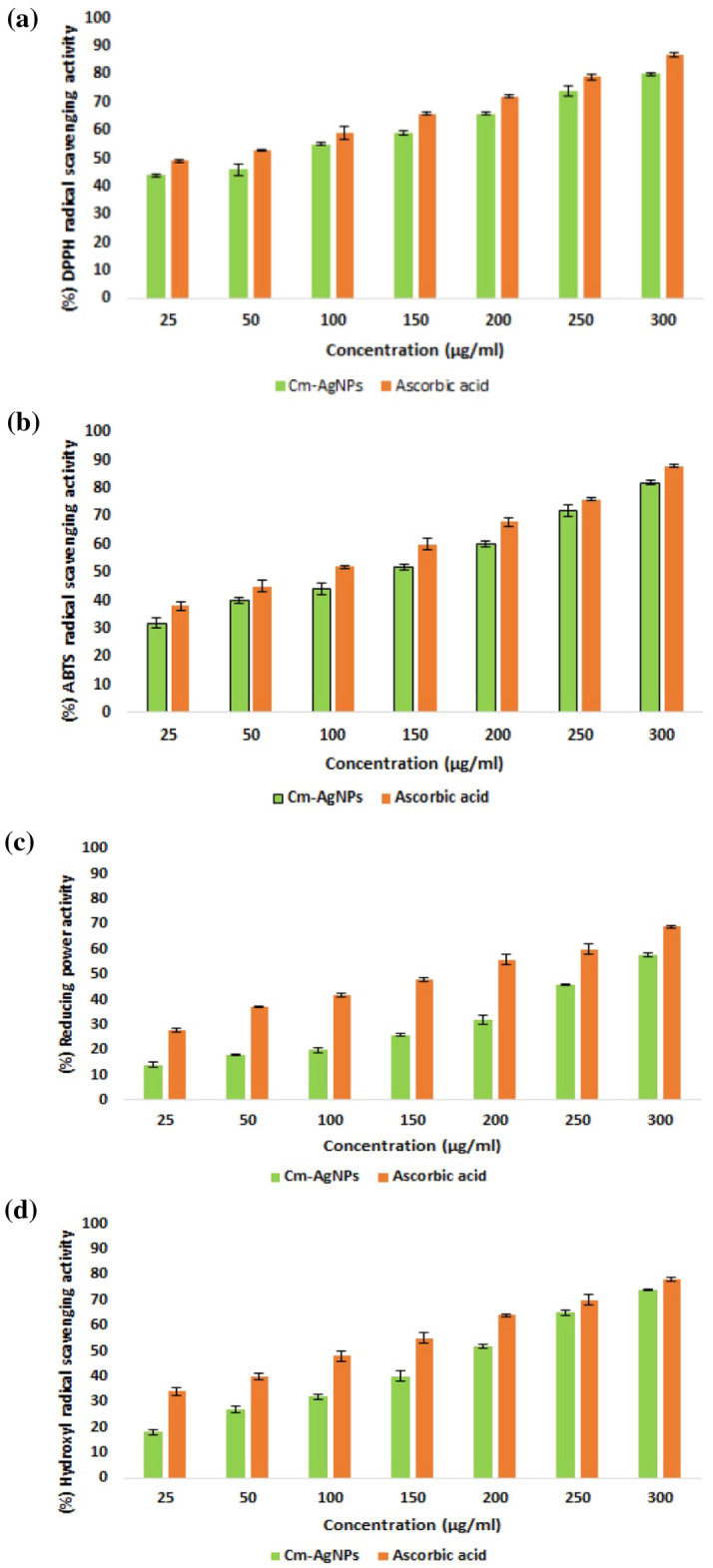
Free radical scavenging ability of Cm-AgNPS and the standard (ascorbic acid) : (**a**) DPPH free radical scavengingactivity (**b**) ABTS + radicals scavenging activity (**c**) reducing power (**d**) hydroxyl-scavenging activity of Cm-AgNPs. Aa-ascorbic; Cm-coconut extract. The significant differences (p ≤ 0.05) shown in the graph are means determined by the analysis of variance (ANOVA) and Tukey’s HSD.

To understand the antioxidant potential of Cm-AgNPs, different antioxidant assays such as DPPH, ABTS, and hydroxyl (OH) radical scavenging activity and reducing power assays were conducted. Various concentrations of Cm-AgNPs in the range of 25, 50, 100, 150, 200, 250, and 300 μg/ml were tested. The present study showed that the antioxidant activity of Cm-AgNPs was dose-dependent, as seen in Fig. 11a–d. The highest test concentration showed maximum radical scavenging activity. Nevertheless, the scavenging activity was lower than that of standard ascorbic acid, as evident in Fig. 11a–d. Cm-AgNPs and ascorbic acid at 300 µg/ml showed maximum scavenging activity of 80% and 87% with the DPPH assay (Fig. 11a). The IC_50_ values recorded for Cm-AgNPs and ascorbic acid were 75.38 ± 0.42 µg/mL and 33.07 ± 0.38 µg/mL. ABTS, reducing power, and hydroxyl scavenging activity at 300 µg/ml were 88%, 69%, and 78%, respectively (Fig. 11b–d).

## Discussion

Green nanotechnology is giving respite to many through its innovative techniques that address several issues and opens new avenues for innovative research explorations in pharmaceutical sciences, agriculture, and drug delivery systems like quantum dots, metallic nanoparticles, organic polymers, and many more^62,63^. Plant-based metal nanoparticles have proven to be biocompatible and safe, and the plethora of signature chemical compounds present in different parts of plants enables them to serve as super reducers, stabilizers, and capping agents during green synthesis^64,65^.

The key indication of productive biosynthesis of nanoparticles is the transformation in the colour of the reaction solution. The nucleation and subsequent growth of NPs as a consequence of the reduction of silver ions to Cm-AgNPs contribute to the broad SPR peak at *λ* max-435 on the UV–Vis spectrum. The peaks between 400 and 500 nm are a validation index for Ag-NP formation^66^. Previous studies on green synthesized NPs have shown peaks at similar positions^15,24^. In addition, the absence of peaks between 500 and 700 nm signifies lack of agglomeration, further reinforcing the stability and small size of green synthesized Cm-AgNPs^67,68^. The peak positions (111, 200, 220, and 311) on the XRD pattern of the Cm-AgNPs explicitly imply that the Cm-AgNPs are face-centered cubic nanocrystals (FCC)^23,69^. Moreover, the peak at 111 in the plane was stronger than the others, indicating that the orientation of nanoparticles was at this position. Consequently, the XRD patterns on the diffractogram validate that the synthesized Cm-Ag-NPs are crystalline without any other phase of impurities. The present findings are consistent with previous reports^3,15,29^.

FTIR analysis of the extract and Cm-AgNPs facilitates identifying the possible secondary metabolites that enable the formation and stabilization of AgNPs. The various peaks on both spectra point towards the presence of moieties of primary and secondary alcohols, carbonyl compounds, lipids, proteins, and aromatic compounds that are vital in reduction, capping, and stability. Carbonyl and amino groups are believed to readily bind to silver, thereby forming a layer around the metal NPs and providing stability^24,30^. The spectra of Cm-AgNPs were closely identical to those of the Cm extract. However, some new peaks, such as 1544 cm^−1^ and 1361 cm^−1^ seen in the spectra of Cm-AgNPs, arose from stretching vibrations of NH and OH amines and alcohols, while the peaks evident at 604 cm^−1^ and 550 cm^−1^ could be attributed to the bending vibrations caused by Ag, which is in agreement with previous studies^6,27,69,70^.] The variations in the position of the peaks in the Cm-AgNPs spectrum imply that the biomolecules present in the Cm-extract have efficiently supported the formation of Cm-AgNPs through the reduction process. Previous studies have reported similar variations in peak positions^10,71^. The FTIR spectra of AgNPs synthesized using coconut inflorescence sap surprisingly showed peaks in similar positions (2990 cm^−1^, 2912 cm^−1^, 1100 cm^−1^); these peaks were attributed to CH_2_ stretching vibration of the pyrrole ring and C–C–CH_2_ linkage of a long carbon^71,72^. Earlier studies have reported coconut meat and coconut water as rich sources of dietary fibers, proteins, amino acids, and alkaloids, which authenticates the present findings^73,74^.

The FESEM-EDS elemental analysis is an important tool that gives insight into the amount of silver and the surface morphology, confirming the successful reduction to stable Ag^0^^69^. The spectrum exhibited peaks with assigned energy levels, and a strong peak at 3 keV is attributable to the silver signal. The chlorine and phosphorus peaks apparent on the spectrum are mainly attributed to the bioactive molecules of coconut extract that get lodged on the AgNPs during nanosynthesis. The carbon signal could be from the mounting material. These findings are comparable to earlier research^30^.

TEM studies show that the Cm-AgNPs were largely spherical and small in size and ranged between 2.01 and 38.76 nm. The data presented is an average calculation of 200 particles (Image J software) plotted by Origen Pro. Silver nanoparticles synthesized from coconut inflorescence sap and leaf extracts measured between 10 and 30 nm, which is in perfect agreement with the present study^72,75^. The average size measured by the zeta sizer showed low PDI but larger particles in comparison to TEM measurements due to the fact that DLS measurements are taken in a liquid state (solution) and include the size of the metallic core, phytochemicals (coatings), and the layer of solution, unlike TEM measurements, which measure solely the metallic core^76^. Similar discrepancies in both readings have been reported earlier, validating our findings^70,76^.

The increase in the number of multidrug resistance microorganisms has raised serious concerns worldwide^77^. Moreover, commercial synthetic medications carry several side effects. This has prompted mankind to explore plant-based alternatives that are milder yet effective against pathogenic microorganisms. The findings of this study show significant inhibition of all the tested pathogenic strains of Candida and bacteria. To our knowledge, there are no studies reported till date that have explored the antimicrobial activity of coconut endosperm AgNPs against pathogenic microorganisms, especially the strains used in this study. The antimicrobial activity showed that bacterial strains were more susceptible than Candida species, which is in agreement with a previous study^78^. But it is noteworthy to mention here that all the NACs used in this study were inhibited, as shown in the in vitro and MIC studies. AgNPs synthesized from different parts of coconut (shell, leaf, inflorescence) were found to be effective against *C. albicans*, *P. aeruginosa, B. subtilis*, *Escherichia coli*, *V.* *parahaemolyticus*, *K. pneumoniae*, *Salmonella typhimurium*, *Listeria monocytogenes,* and *Citrobacter freundii.* The aforementioned strains showed a ZOI between 12.44 and 22.12 mm, and the MIC and MFC were between 13.6 and 1000 µg/ml, which is consistent with the present findings^75,79,80^. Besides, coconut oil also exhibited antimicrobial activity in a previous study^81^. Earlier research has shown the robust anticandidal activity of biogenic silver nanoparticles against a wide array of Candida species, including *C. albicans*, *C. glabrata*, *C. tropicalis*, *C. krusei,* and *C. dubliniensis,* with MIC and MFC values (62–1000 µg/ml)^78,82^, which are quite comparable to our findings. Similarly, congruent with the present study, biosynthesized AgNPs have displayed enhanced antibacterial activity against a panel of several bacterial strains^30,75,77,83^.

The discrepancies in the in vitro inhibition shown by bacterial and fungal strains could be due to the differences in the cell structural organization, chemical composition of the cell wall, and cell membrane. Additionally, bacterial cells, being prokaryotes, are less complex than fungal cells, allowing easy permeation and resulting in stronger inhibitory activity. On the contrary, fungal cells have inert cell structures like the cell wall and its components, which are considered major barriers with a superior detoxification system^84^. However, to our knowledge, there are no prior studies to compare the effect of AgNPs synthesized from coconut meat against the microorganisms used in this study, especially against Candida spp.

The findings of in vitro antimicrobial activity of Cm-AgNPs were further substantiated by SEM studies. The Cm-AgNPs at their MIC concentrations induced structural alterations in both Candida and bacterial spp., and a clear demarcation was seen between the treated and untreated cells. Cells treated with NPs displayed exfoliation, ruggedness, and cell leakage, which were evident during scanning and are also depicted in microphotographs. In accordance with the findings of the present study, earlier investigations showed agglomerated AgNPs surrounding severely damaged cells of *C. auris*, *C. glabrata*, *C. albicans*, and *C. parapsilosis, when treated* with biosynthesized AgNPs^85,86^.

Various researchers have put forth their views regarding the mode of action of AgNPs against Candida and bacterial spp.; however, it is an accepted fact that AgNPs target the cell wall and cell membrane, thereby altering their permeability^87,88^. The shape, size, and surface chemistry of the biosynthesized NPs influence their antimicrobial properties. Although the exact mechanism of action still needs precision. The microbial cell wall plays a crucial role in providing rigidity and maintaining the cellular structure by balancing osmotic concentration. The AgNPs are competent at destructing the lipid bilayer and disrupting the cell membrane integrity and permeability, consequently leading to the leakage of ions, which results in disturbed electrical potential of the membrane and the formation of pores^15,89^. Therefore, cell membranes get distorted or damaged, which is evident in the findings of SEM in this study. Additionally, the AgNPs block the G2/M phase of the cell cycle^90^. It is also reported that AgNPs inhibit β-glucan synthase and budding due to damage to the cell surface, which could lead to cell disruption^90^. The AgNPs trigger concurrent alterations in the structure and metabolism of the cells by changing the cell surface morphology and impacting the metabolic functions of Candida cells by increasing ROS production^91^, membrane depolarization^92^, nuclear fragmentation, promoting inhibition of enzyme function, and causing cell arrest^92,93^. Besides, AgNPs also cause dysfunctional apoptosis of the mitochondria and damage the cellular ergosterol in Candida spp., as well as programmed cell death due to the accumulation of ROS^91,92^.

The mode of action of AgNPs against bacterial strains is quite similar to the antifungal mechanism. Once inside the cell cytoplasm, the effects of silver ions or silver nanoparticles on the cell metabolism are similar to the events described above for anticandidial activity. Another view is that AgNPs have a special affinity for the thiol sulfhydryl group present in the cell membrane of bacterial cells^87^. AgNPs, after adherence, release silver ions, which replace the H + cation of the thiol group present in the cell membrane to form an S–Ag group. The formation of S–Ag triggers an alteration in cell permeability, leading to a series of events like an enhanced influx of silver ions, protein leakage, and dissociation of the cell surface membrane, which instigates the formation of pores or holes, eventually culminating in cell death^6,75,76^. The deformed cell surface, holes, depressions, and cell leakage seen in the microphotographs of the present study could therefore be from the disturbed cell permeability and the subsequent damage caused by silver ions or NPs to the cell structure.

The phytochemical analysis in this study shows the presence of phytoconstituents that have also been reported earlier^48,73,94^. In a previous study, phytochemical analysis of the aqueous extract of coconut endosperm showed tannins, saponins, resins, and alkaloids^73,94^. Another investigation also showed alkaloids, terpenoids, phenols, and saponins in the cotyledons of coconut and inflorescence^48,72^. Previous reports highlight the fact that AgNPs are surrounded by a fine layer of organic molecules, which substantiates the role of phytochemicals as capping ligands^30,73^. The FTIR spectrum also revealed similar bioactive molecules, which confirms the phytochemical findings.

The various antioxidant assays displayed the potent antioxidant activity of Cm-AgNps in a dose-dependent manner. The results of DPPH assays were quite comparable to ABTS, reducing power, and OH scavenging assays with minor differences, which is in agreement with previous antioxidant studies involving phytofabricated AgNPs^70,76^. The DPPH and ABTS radical scavenging activity of young and mature coconut meat and water ranged between 32 and 91%^73^; coconut cotyledon extracts exhibited 88% antioxidant activity at 1000 mg/ml^48^. These studies are in agreement with the present findings. A possible explanation for the antioxidant potential of AgNPs was credited to the diverse phytochemicals present in the plant extract^95,96^. Phytochemicals, especially polyphenols, are excellent reactive oxygen species (ROS) scavengers due to the hydroxyl groups, aromatic rings, and their efficient conjugate systems that can nullify the ROS effects by breaking the radical chain and metal chelation^97^. Significant free radical scavenging properties and reducing power capacity are linked to alkaloids, tannins, and saponins, which have an excellent ability to quench ROS by acting as oxidizing and reducing agents^76,98^. Hence, the antioxidant activity of Cm-AgNPs could be credited to the polyphenols and alkaloids present in the Cm-extract. The bioactive molecules get lodged on the surface of the AgNPs during synthesis and serve as capping agents. Based on the findings of antioxidant assays, it can be deduced that the Cm-AgNPs served as efficient antioxidants, and the antioxidant compounds present in the Cm-extract contributed to their activity.

The excellent antimicrobial properties shown by Cm-AgNPs can therefore be attributed to their morphology (size and shape) and surface chemistry, as these properties are vital during the interaction of NPs with microbial cells. The enhanced antimicrobial activity of NPs was credited to their small size, which enables efficient adherence to the microbial cell membrane, easing the process of penetration^99^. Furthermore, the antioxidant activity displayed by Cm-AgNPs could be due to the important phytochemicals that form a layer around the NPs, defining their enhanced antioxidant ability. Earlier studies have also highlighted the role of phytochemicals in the capping, stabilization, antimicrobial, and antioxidant properties of NPs^31,100^.

Although nanoparticles have been used in a broad range of applications in various sectors like medicine, agriculture, public health, and industry. However, the toxic effects and negative impact of exposure to nanoparticles on the environment and human health cannot be ignored^101^. Hence, a deep understanding of the underlying mechanisms and more research studies to minimize the risks with precise utilization are essential for sustainable development.

## Conclusion

This study focused on the facile and eco-friendly route to synthesize AgNPs from coconut meat (Cm). The phytofabrication was confirmed with UV–Vis. The characterization of Cm-AgNPs with XRD validated the crystallinity of the NPs. FTIR revealed the phytomolecules that aided in capping. The elemental composition, morphology, size, and surface charge were studied with EDS, SEM, TEM, and DLS. The synthesized nanoparticles were evaluated against a panel of multidrug-resistant strains of bacteria and Candida. The NPs exhibited potent antimicrobial activity, which was confirmed by SEM studies. The SEM showed severely damaged, deformed, and ruptured cells at their MIC concentrations. Phytochemical analysis of Cm-extract showed phytoconstituents that contributed to capping. Additionally, the Cm-AgNPs demonstrated significant antioxidant activity. Based on the findings of this study, it can be concluded that Cm-AgNPs can serve as an excellent therapeutic agent against pathogenic microorganisms, especially the emerging multi-drug-resistant strains including *Candida auris, S. epidermidis, and MRSA*. However, in vivo experimental studies to understand the potential toxicity and biocompatibility of Cm-AgNPs are needed. We recommend future work to understand the mechanism of microbe–nanoparticle interactions in order to prevent nanoparticle resistance.

## Data Availability

The data used in this study are available from the corresponding author upon request.
